# α-Chaconine Affects the Apoptosis, Mechanical Barrier Function, and Antioxidant Ability of Mouse Small Intestinal Epithelial Cells

**DOI:** 10.3389/fpls.2021.673774

**Published:** 2021-06-09

**Authors:** Yuhua He, Jiaqi Chen, Qiyue Zhang, Jialong Zhang, Lulai Wang, Xiaoxia Chen, Adrian J. Molenaar, Xuezhao Sun

**Affiliations:** ^1^College of Animal Science and Technology, Jilin Agricultural Science and Technology University, Jilin City, China; ^2^The Innovation Centre of Ruminant Precision Nutrition and Smart and Ecological Farming, Jilin Agricultural Science and Technology University, Jilin City, China; ^3^Jilin Inter-Regional Cooperation Centre for the Scientific and Technological Innovation of Ruminant Precision Nutrition and Smart and Ecological Farming, Jilin City, China; ^4^AgResearch Ltd., Grasslands Research Centre, Palmerston North, New Zealand

**Keywords:** α-chaconine, intestinal epithelial cell, cell cycle, cell apoptosis, mechanical barrier function, antioxidant ability, mouse

## Abstract

α-Chaconine is the most abundant glycoalkaloid in potato and toxic to the animal digestive system, but the mechanisms underlying the toxicity are unclear. In this study, mouse small intestinal epithelial cells were incubated with α-chaconine at 0, 0.4, and 0.8 μg/mL for 24, 48, and 72 h to examine apoptosis, mechanical barrier function, and antioxidant ability of the cells using a cell metabolic activity assay, flow cytometry, Western blot, immunofluorescence, and fluorescence quantitative PCR. The results showed that α-chaconine significantly decreased cell proliferation rate, increased apoptosis rate, decreased transepithelial electrical resistance (TEER) value, and increased alkaline phosphatase (AKP) and lactate dehydrogenase (LDH) activities, and there were interactions between α-chaconine concentration and incubation time. α-Chaconine significantly reduced the relative and mRNA expressions of genes coding tight junction proteins zonula occludens-1 (ZO-1) and occludin, increased malondialdehyde (MDA) content, decreased total glutathione (T-GSH) content, reduced the activities of superoxide dismutase (SOD), catalase (CAT), glutathione peroxidase (GSH-Px), and γ-glutamylcysteine synthetase (γ-GCS) and the mRNA expressions of SOD, CAT, GSH-Px, and γ-GCS genes. In conclusion, α-chaconine disrupts the cell cycle, destroys the mechanical barrier and permeability of mucosal epithelium, inhibits cell proliferation, and accelerates cell apoptosis.

## Introduction

Potato is a major crop used as food, animal feed, and industrial raw materials and is grown in about 80% of the countries in the world (Ahmad et al., 2019). Potatoes contain glycoalkaloids, and the content of glycoalkaloids is especially high when exposed to light or to high temperatures during storage (Machado et al., 2007; Shepherd et al., 2016). Glycoalkaloids have a bitter taste, which affects palatability, and are toxic (Iablokov et al., 2010). Furthermore, glycoalkaloids are resistant to high temperatures and are difficult to remove or destroy (Liu et al., 2020). Although crop breeders are making progresses in reducing the content of glycoalkaloids through molecular breeding (Yasumoto et al., 2019), this is a long-term goal, and glycoalkaloids are still an important factor affecting the utilization of potatoes and their by-products.

**FIGURE 1:**
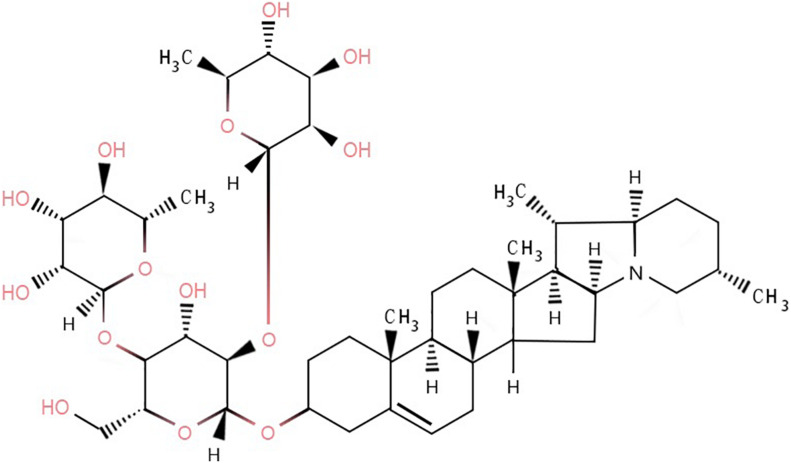
Molecular structure of α-chaconine.

In the case of normal maturity and no exposure to light, the content of glycoalkaloids in potatoes is generally 10–100 mg/kg of fresh weight (Knuthsen et al., 2009). There are six main types of glycoalkaloids in potatoes, of which α-solanine and α-chaconine are the most abundant (Milner et al., 2011), accounting for 95% of the total content (Nielsen et al., 2020), with α-chaconine being the most abundant (Rytel, 2012; Tajner-Czopek et al., 2012). The ratio of α-solanine to α-chaconine is approximately 1:2 to 1:7 (Skarkova et al., 2008). α-Chaconine is a cyclopentane phenanthrene compound (Oda et al., 2002), which links the nitrogen-containing steroid group with 1–4 monosaccharides through a 3-O-glycosidic bond. α-Chaconine includes one molecule of D-glucose and two molecules of L-rhamnose (Figure 1).

The toxicity of glycoalkaloids such as α-chaconine to animals is mainly via the perturbation of the gastrointestinal tract. The clinical signs of glycoalkaloid toxicity of potatoes include dry mouth, pain in the mouth and throat, tightening of the throat, nausea, vomiting, abdominal pain, and diarrhea (Korpan et al., 2004; Lin et al., 2018). Severe vomiting and diarrhea can lead to electrolyte imbalance, dehydration, and hypotension. In addition, glycoalkaloid toxicity can affect the cardiovascular and reproductive systems.

Intestinal mucosal epithelial cells have barriers such as tight junctions as well as the cell membrane preventing the invasion of harmful microorganisms and certain toxins from penetrating the intestine. Some anti-nutritional factors or toxins directly act on intestinal mucosal epithelial cells to affect cell proliferation, accelerate cell apoptosis, and thereby destroy the mechanical barrier function of the intestine (Groschwitz and Hogan, 2009; Zhao et al., 2017). Reactive oxygen species (ROS) can also directly act on intestinal epithelial cells, causing lipid peroxidation of the phospholipid layer in the cell membrane, destroying the membrane structure of epithelial cells and inducing apoptosis (Omatsu et al., 2009). This ultimately reduces the mechanical barrier function of intestinal epithelial cells and allows many toxic components to enter the intestinal tract.

It has been reported that α-chaconine is a highly cytotoxic glycoalkaloid in potatoes (Oda et al., 2002; Friedman, 2006; Ji and Gao, 2008). However, how α-chaconine affects the small intestinal mucosal epithelial cells is not clear. We speculate that the toxicity of α-chaconine to the digestive system may be due to its destruction of the mechanical barrier function of the intestine or oxidative damage to the intestine. Therefore, in this study, mouse small intestinal epithelial cells were used as a model to study the effects of α-chaconine on cell proliferation, cell cycle, apoptosis, mechanical barrier function, and antioxidant ability using *in vitro* cell culture. The exploration of the toxicity of α-chaconine to digestive system cells at the molecular level would provide solid scientific data for revealing the mechanisms of how α-chaconine affects intestinal health.

## Materials and Methods

### Cell Culture

The murine intestinal epithelial cell line MODE-K (Shanghai Jining Industrial Co., Ltd., Shanghai, China) was adopted for the study. The cells were washed with a cell culture solution that was comprised of the 1640 medium (PM150910; Procell, Wuhan, China) +10% fetal bovine serum (FBS, Gibco, Carlsbad, NM, United States) solution (pH 7.2–7.4) +1% penicillin-streptomycin (Sigma, St. Louis, MO, United States) to remove DMSO. The resuscitated cells were cultured in a fresh cell culture solution and under 5% CO_2_ saturated humidity at 37°C. The cell culture experiments were performed in triplicate with α-chaconine treatment at concentrations of 0, 0.4, and 0.8 μg/mL. α-Chaconine stock solution (16 mg/100 mL) was made and diluted with the cell culture solution. The choice of the concentrations was based on pilot trials. Measurements were performed in triplicate after incubation for 24, 48, and 72 h.

### Measurements of Cell Proliferation, Cell Cycle, and Apoptosis

#### Cell Proliferation

The MODE-K cells are regarded as well grown when their morphology becomes oval or polygonal in shape, in a monolayer adherent to the plate wall without overlapping, and in the arrangement of paving stones. Cells from the same generation were seeded on 96-well cell culture plates. When the cells reached confluence, α-chaconine (Shanghai Yuanye Bio-Technology Co., Ltd., Shanghai, China) was added at the designated concentrations. Cell proliferation rate was measured using the MTT [3-(4,5-dimethylthiazol-2-yl)-2,5-diphenyltetrazolium bromide] method (Kumar et al., 2018) when cells were harvested. The cells harvested in the logarithmic growth phase were adjusted to a cell density of 5 × 10^4^/ml using the 1640 medium. The cell suspension (100 μL) together with sterile phosphate buffered saline (PBS; 100 μL) was transferred to a 96-well cell culture plate and incubated overnight at 37°C. Then 10 μL of MTT (Hefei Labgic Technology Co., Ltd., Hefei, China) was added to the cells and incubated at 37°C for further 4 h. After the medium was removed and 150 μL DMSO (dimethyl sulfoxide; Solarbio, Shanghai, China) was added, the plate was shaken for 10 min and the absorbance was measured at OD 568.

#### Cell Cycle

MODE-K cells in their logarithmic growth phase were adjusted to a cell density of 1.5 × 10^5^/mL with the 1640 medium. The cell suspension solution was transferred into six-well plates with 2 mL each well and cultured overnight at 37°C. At the end of incubation, the cells were digested with 2 mL of 0.25% trypsin (without EDTA) for 1–2 min. Once the cells were separated from each other, centrifugation was performed at 221 × *g* for 5 min (Eppendorf model 5702R, Hamburg, Germany) to remove the supernatant. Then the cells were resuspended with the PBS buffer and centrifuged as described above. This procedure was repeated and 700 μL of pre-cooled 80% ethanol was slowly added to the pellet to make the final ethanol concentration of 70%. After fixing in ethanol at 4°C for at least 4 h, the cells were centrifuged at 221 × *g* for 5 min and washed with pre-cooled PBS buffer and collected by centrifugation twice. The cells were incubated at 37°C for 30 min in 100 μL of RNase (50 μg/mL) and then stained with 100 μL of propidium iodide (50 μg/mL) at 4°C for 30 min in the dark. The stained cells were tested for their stage of cell cycle using flow cytometry (BD Accuri^TM^ C6 flow cytometer, BD Biosciences, San Jose, CA, United States) (Kim and Sederstrom, 2015).

#### Cell Apoptosis

After the cells were treated with 0, 0.4, or 0.8 μg/ml α-chaconine for 24, 48, or 72 h, cell apoptosis was detected with an Annexin V-FITC/PI apoptosis detection kit (KGA108, Jiangsu KeyGEN BioTECH Co., Ltd., Nanjing, China) according to the manufacturer’s instructions. The stained samples were quantified at 488 nm (emission wavelength) and 470 nm (excitation wavelength) using a high-sensitive flow cytometer (Beckman Coulter CytoFLEX S, Krefeld, Germany).

### Permeability and Structural Integrity of MODE-K Cells

The transepithelial electrical resistance (TEER) values of the MODE-K cells after 24, 48, and 72 h of incubation with α-chaconine were measured using a Millicell ERS-2 V-Ohm Meter (MERS00002, EMD Millipore Ltd., Watford, United Kingdom). Measurements were conducted in triplicate at three points in different directions in each Transwell (Corning^®^ Transwell polycarbonate membrane inserts, Cat No. 3422, 8 μm pore size; 6.5 mm membrane diameter, Corning Incorporated, Tewksbury, MA, United States) according to the manufacture’s instructions. The measured datum multiplied by the Transwell membrane area (0.6 cm^2^) gave the TEER value of the sample in units of Ω × cm^2^ (Pan et al., 2013). The activities of alkaline phosphatase (AKP) and lactate dehydrogenase (LDH) in cells were measured according to the kit instructions (Nanjing Jiancheng Bioengineering Research Institute, Nanjing, China).

### Distribution and Relative Expression of Tight Junction Proteins in MODE-K Cells

The distribution of tight junction proteins zonula occludens-1 (ZO-1) and occludin were determined using immunofluorescence staining (Im et al., 2019). MODE-K cells were grown on glass slides and treated with 0, 0.4, or 0.8 μg/mL α-chaconine for 24, 48, or 72 h. After washing with PBS three times for 3 min each time, the cells were fixed with 4% paraformaldehyde for 15 min at room temperature and washed again as previously described. The cells were then permeabilized with 0.5% Triton X-100 (made with PBS) for 20 min. After washing as before and the medium removed, the cells were blocked with goat serum for 30 min at room temperature. After the blocking solution was removed by drawing off with paper tissue, the cells were incubated with rabbit anti-claudin (59 KD; diluted 1:100; 13409-1-AP) and anti-ZO-1 (230 KD; diluted 1:100; 21773-1-AP) antibodies (Wuhan Sanying Biotechnology Ltd., Wuhan, China) overnight at 4°C. The cells were washed three times with PBST (phosphate buffered saline Tween-20) and incubated with Cy3-conjugated secondary antibody goat anti-rabbit IgG (diluted 1:100; CWBIO, Beijing, China) for 2 h at 37°C. For staining, after three washes with PBST, DAPI (4’,6-diamidino-2-phenylindole) was added and incubated for 5 min in the dark. The stained cells were washed thoroughly with PBST four times for 5 min. After the slides were sealed with a slide containing an anti-fluorescence quenching agent, images were captured using a fluorescence microscope (Olympus BX53; Olympus, Center Valley, PA, United States) and attached a camera (Olympus DP72; Olympus, Center Valley, PA, United States).

The relative levels of tight junction proteins ZO-1 and occludin in MODE-K cells were determined using the Western Blot method (Pan et al., 2013). After the removal of the culture medium, the α-chaconine-treated cells were washed with 10 mM PBS (pH 7.2–7.3) three times. Proteins were extracted from the cells with PMSF (ST506, Beyotime Biotechnology, Shanghai Ltd., China)-containing cell lysate (P0013B; Beyotime Biotechnology Ltd., Shanghai, China). The extracted proteins were determined for total protein content using a BCA assay kit (P0010; Beyotime Biotechnology Ltd., Shanghai, China). After the preparation of 5–12% sodium dodecyl sulfate polyacrylamide gel electrophoresis (SDS-PAGE), 15 μL of cell protein extracts (1 mg/mL) was loaded for separation. The separated proteins were transferred onto nitrocellulose using the semidry transfer film method. The membranes were blocked in 3% non-fat milk/TBST (tris buffered saline Tween-20) (pH 7.6) at room temperature for 2 h and then incubated with polyclonal rabbit anti-claudin (59 KD; diluted 1:1000; 13409-1-AP; Wuhan Sanying Biotechnology Ltd., Wuhan, China), polyclonal rabbit anti-ZO-1 (230 KD; diluted 1:800; 21773-1-AP; Wuhan Sanying Biotechnology Ltd., Wuhan, China), or monoclonal mouse anti- β-actin (42 KD; diluted 1:200; BM0627; Boster Biological Technology Co., Ltd., Wuhan, China) antibodies diluted in TBST buffer overnight at 4°C. After washing with the TBST buffer 5–6 times for 5 min, the membranes were incubated with goat anti-rabbit IgG HRP-conjugated secondary antibodies (diluted 1:50000, BA1054; Boster Biological Technology Co., Ltd., Wuhan, China) for 1.5 h at 37°C. The membranes were washed with TBST buffer 5–6 times for 5 min and visualized for immunoreactive bands using chemiluminescence with an ECL detection kit (Santa-Cruz Biotechnology, Santa Cruz, CA, United States). The bands were scanned for gray levels and quantification was performed using Quantity One software with the integrated density value of β-actin for normalization.

### mRNA Expressions of Tight Junction Protein Genes ZO-1 and Occludin and Oxidase Genes SOD, CAT, GSH-Px, and γ-GCS Using qPCR

The total RNA was extracted using the Aidlab kit (Aidlab Biotechnologies Co., Ltd., Beijing, China) in the TRIzol method (Heidary and Kakhki, 2014) and stored at −80°C for later use. RNA was reverse transcribed into cDNA using the common reverse primer Oligo (dT) with a cDNA synthesis kit (Vazyme Biotech Co., Piscataway, NJ, United States). The reverse transcription reaction system is shown in Table 1. The cDNA was diluted 10-fold and detected using the real-time fluorescence quantitative PCR detection technique. The reaction system is shown in Table 2. The primers were designed and synthesized by Wuhan Biofavor Biotech Services Co. Ltd. (Wuhan, China) and are listed in Table 3. The quantification of mRNA expression of genes was made with β-actin as the internal reference gene.

**TABLE 1:** Reverse transcription reaction.

Regent	Volume (μL)
RNA	1–5 μg
Oligo (dT) 18 (10 μM)	2
dNTP (2.5 mM)	4
5 × Hiscript buffer	4
Hiscript reverse transcriptase	1
Ribonuclease inhibitor	0.5
ddH_2_O (RNase free)	up to 20

*Reaction conditions: 25°C for 5 min, 50°C for 15 min, 85°C for 5 min, and 4°C for 10 min.*

**TABLE 2:** Real time fluorescence quantitative PCR reaction.

Regent	Volume (μL)
cDNA (10-fold dilution)	2
Forward primer (10 μM)	1
Reverse primer (10 μM)	1
SYBR green master mix	5
50 × ROX reference dye 2	0.2
H_2_O	0.8

*Reaction conditions: 50°C for 2 min, 95°C for 10 min, 95°C for 30 s, 60°C for 30 s, 40 cycles. The final data were analyzed using 2^–ΔΔCt^.*

**TABLE 3:** Primer sequence.

Name	Primer	Sequence	Size (bp)
β-actin	Forward	5′-CACGATGGAGGGGCCGGACTCATC-3′	240
	Reverse	5′-TAAAGACCTCTATGCCAACACAGT-3′	
Mus occludin	Forward	5′-TAAGAGCTTACAGGCAGAACTAG-3′	228
	Reverse	5′-CTGTCATAATCTCCCACCATC-3′	
Mus ZO-1	Forward	5′-AGATACCTGTGAACTGTCCCTA-3′	253
	Reverse	5′-ATGTCCGCACCTGAGTGA-3′	
Mus SOD	Forward	5′-AACCATCCACTTCGAGCAGA-3′	203
	Reverse	5′-GGTCTCCAACATGCCTCTCT-3′	
Mus GSH-Px	Forward	5′-CAGAATGGCAAGAATGAAGAG-3′	132
	Reverse	5′-GAAGGTAAAGAGCGGGTGA-3′	
Mus CAT	Forward	5′-GATGAGCGGGCTACCTTA-3′	206
	Reverse	5′-TGTGGAGACTGCGTGGAA-3′	
Mus γ-GCS	Forward	5′-ACAAGCACCCCCGCTTCGG-3′	140
	Reverse	5′-CTCCAGGCCTCTCTCCTC-3′	

### Antioxidant Ability of MODE-K Cells

The levels of malondialdehyde (MDA) and total glutathione (T-GSH) and the enzyme activities of superoxide dismutase (SOD), catalase (CAT), glutathione peroxidase (GSH-Px), and γ-glutamylcysteine synthetase (γ-GCS) were all determined using test kits (Nanjing Jiancheng Bioengineering Research Institute, Nanjing, China) in accordance with corresponding instructions.

### Statistical Analysis

Triplicate readings for each measurement were averaged before statistical analysis. The data for statistical analysis were put to the general linear model with α-chaconine concentration, incubation time, and their interaction as the fixed effects and incubation time as the repeated measurement and analyzed using SPSS19.0 software. The Ducan’s multiple comparison test was used to determine statistical differences among the means. Probability levels below 0.05 were considered statistically significant.

## Results

### Mouse Intestinal Epithelial Cell Proliferation, Cell Cycle, and Apoptosis

α-Chaconine concentration and incubation time had statistically significant effects on the proliferation, cell cycle, and apoptosis of mouse intestinal epithelial cells (Table 4).

**TABLE 4:** Effects of α-chaconine on the proliferation, cell cycle, and apoptosis of mouse intestinal epithelial cells.

Concentration (μg/mL)	Time (h)	Cell proliferation rate (%)	Cell cycle distribution (%)			Apoptosis rate (%)
			G_0_/G_1_	S	G_2_/M	
0	24	100.7^a^	41.5^i^	46.9^a^	11.6^b^	3.9^f^
	48	103.2^a^	44.1^h^	42.3^b^	13.5^a^	5.2^ef^
	72	103.2^a^	47.0^g^	42.0^b^	11.0^b^	5.8^e^
0.4	24	95.2^b^	48.5^f^	41.3^b^	10.2^b^	7.7^d^
	48	90.8^c^	52.6^e^	36.0^c^	11.4^b^	10.2^c^
	72	81.9^de^	61.3^c^	31.5^e^	7.2^c^	11.2^c^
0.8	24	84.1^d^	56.4^d^	32.9^d^	10.6^b^	15.1^b^
	48	78.6^e^	67.3^b^	28.6^f^	4.2^d^	15.7^b^
	72	70.6^f^	73.4^a^	25.2^g^	1.4^e^	35.7^a^
SEM		0.04	3.41	2.29	1.25	3.06
*P-*value	Conc	<0.001	<0.001	<0.001	<0.001	<0.001
	Time	<0.001	<0.001	<0.001	<0.001	<0.001
	Conc × Time	<0.001	<0.001	<0.001	<0.001	<0.001

*The same letter on the shoulder of the mean in the same column indicates that the difference is insignificant, and the absence of the same letter means that the difference is significant. Conc, concentration.*

The cell proliferation rate did not change significantly over time in the control (0 μg/mL) cells, however, it significantly decreased with increasing incubation time in the 0.4 and 0.8 μg/mL groups. The cell proliferation rate significantly decreased with increased α-chaconine concentration at the same incubation time.

The G_0_/G_1_ ratio significantly increased with incubation time in all treatments and with α-chaconine concentration. The proportion of cells in the S stage of the cell cycle was less by 10% (*P* < 0.001) at 48 h of incubation than at 24 h and did not further decrease (*P* > 0.05) until 72 h for the control group. The proportion also significantly decreased with incubation time for the α-chaconine-treated groups and the extent of decrease was similar (*P* > 0.05) for the two α-chaconine-treated groups. The average decrease was 13% (*P* < 0.001) at 48 h and 24% (*P* < 0.001) at 72 h compared with 24 h. The G_2_/M ratio in the control group first increased and then decreased with time. When α-chaconine was added at 0.4 μg/mL, the G_2_/M ratio did not change until 48 h (*P* > 0.05) but was significantly lower at 72 h than at 24 and 48 h of incubation. When the α-chaconine concentration was increased to 0.8 μg/mL, the G_2_/M ratio significantly decreased with incubation time. At 24 h of incubation, α-chaconine concentration had no significant effect on G_2_/M ratios, however, at 48 and 72 h, the G_2_/M ratio significantly decreased with the concentration.

The apoptosis rate was significantly higher at 72 h than at 24 h in the untreated cells, higher at 48 and 72 h than at 24 h in the 0.4 μg/mL treated cells, and higher at 72 h than at 24 and 48 h in the 0.8 μg/mL treated cells. When a comparison was made for different α-chaconine concentrations at the same incubation time, the apoptosis rate was always related with α-chaconine concentration.

### Mechanical Barrier Function of MODE-K Cells

#### Permeability and Structural Integrity of MODE-K Cells

The concentration of α-chaconine and time of incubation had significant effects on TEER values, and AKP and LDH activities in mouse intestinal epithelial cells (*P* < 0.05; Table 5).

**TABLE 5:** Effects of α-chaconine on the permeability and structural integrity of mouse intestinal epithelial cells.

Concentration (μg/mL)	Time (h)	Cell TEER value (Ω cm^2^)	AKP (King’s unit/100 mL)	LDH (U/L)
0	24	93.3^a^	0.691^d^	755.1^e^
	48	93.0^a^	0.670^d^	780.4^e^
	72	94.5^a^	0.661^d^	760.7^e^
0.4	24	86.2^ab^	0.725^d^	909.5^e^
	48	82.9^b^	0.888^bc^	1173.3^d^
	72	73.9^c^	0.903^bc^	1369.8^bc^
0.8	24	73.6^c^	0.866^c^	1215.4^cd^
	48	51.5^d^	0.966^ab^	1468.1^b^
	72	18.7^e^	1.028^a^	1746.0^a^
SEM		7.81	0.0430	111.57
*P-*value	Conc	<0.001	<0.001	<0.001
	Time	<0.001	<0.001	<0.001
	Conc × Time	<0.001	<0.001	<0.001

*The same letter on the shoulder of the mean in the same column indicates that the difference is insignificant, and the absence of the same letter means that the difference is significant. TEER, transepithelial electrical resistance; AKP, alkaline phosphatase; LDH, lactate dehydrogenase; Conc, concentration.*

The TEER value of the control cells did not change significantly with the prolongation of incubation time (*P* < 0.05) but was significantly lower at 72 h than at 24 and 48 h in the 0.4 μg/mL treated cells and decreased significantly with incubation time in the 0.8 μg/mL treated cells.

At 24 h, the TEER value in the 0.8 μg/mL group was significantly lower than that of the control and 0.4 μg/mL treated cells. At 48 and 72 h, the TEER value was significantly decreased with the increased concentration of α-chaconine.

There was no significant change in AKP activity in the control cells with incubation time. However, the activity increased significantly (*P* < 0.05) from 24 h to 48 h of incubation time in the 0.4 and 0.8 μg/mL treated cells, but did not significantly increase from 48 h to 72 h. α-Chaconine started to have an effect at 48 h after incubation for the 0.4 μg/mL treatment group and at 24 h for the 0.8 μg/mL treatment compared with the 0 μg/mL control treatment.

The LDH activity in the control cells did not change significantly with incubation time but significantly increased with incubation time in the 0.4 and 0.8 μg/mL treated cells. Similarly to the AKP activity response, α-chaconine started to have an effect (*P* < 0.05) on LDH activity from 24 h of incubation at the concentration of 0.8 μg/mL in comparison with the control. However, with the 0.4 μg/mL concentration 48 h incubation was needed to have a detectable effect (*P* < 0.05) relative to the control.

#### Distribution and Relative Expression of Tight Junction Proteins in MODE-K Cells

α-Chaconine concentration and incubation time had significant effects on the average immunofluorescence staining of tight junction proteins ZO-1 and occludin and the relative expression of tight junction protein ZO-1 in mouse intestinal epithelial cells, but the effect on the relative expression of the tight junction protein occludin did not reach statistical significance (Table 6 and Supplementary Figure 1).

**TABLE 6:** Effects of α-chaconine on the distribution and relative expression of tight junction proteins in mouse intestinal epithelial cells.

Concentration (μg/mL)	Time (h)	ZO-1 (average optical density)	Occludin (average optical density)	ZO-1/β -actin	Occludin/β -actin
0	24	0.852^a^	0.900^a^	0.585^a^	0.537^a^
	48	0.885^a^	0.838^a^	0.594^a^	0.558^a^
	72	0.897^a^	0.850^a^	0.568^a^	0.550^a^
0.4	24	0.694^b^	0.738^b^	0.553^a^	0.473^ab^
	48	0.644^b^	0.576^c^	0.447^b^	0.403^bc^
	72	0.583^c^	0.503^cd^	0.321^c^	0.286^cd^
0.8	24	0.549^c^	0.534^cd^	0.436^b^	0.354^bcd^
	48	0.489^d^	0.463^d^	0.288^c^	0.249^de^
	72	0.415^e^	0.309^e^	0.176^d^	0.134^e^
SEM		0.0562	0.0640	0.0474	0.0475
*P-*value	Conc	<0.001	<0.001	<0.001	<0.001
	Time	0.002	<0.001	<0.001	0.050
	Conc × Time	0.002	0.021	0.013	0.104

*The same letter on the shoulder of the mean in the same column indicates that the difference is insignificant, and the absence of the same letter means that the difference is significant. Conc: concentration.*

The average protein expression level of ZO-1 as measured by immunofluorescence staining was stable over time in the control cells, while it decreased with time and a significant difference was obtained at 72 h in the 0.4 μg/mL treated cells compared with the control cells, and at all incubation times in the 0.8 μg/mL treated cells. The effects of α-chaconine concentration and incubation time on the average immunofluorescence staining levels of occludin were similar to those on the immunofluorescence staining levels of ZO-1.

The relative expression of tight junction proteins ZO-1 and occludin in the control cells remained similar over time but decreased with time in α-chaconine treatments. The effects of α-chaconine at 4 μg/mL were not significant at 24 h compared with 0 μg/mL but were significant (*P* < 0.05) from 48 h of incubation. At 0.8 μg/mL, α-chaconine had significant effects (*P* < 0.05) starting from 24 h of incubation. The effect of α-chaconine was stronger (*P* < 0.05) at 48 and 72 h of incubation time when the concentration increased from 0.4 to 0.8 μg/mL.

#### mRNA Expression of Tight Junction Protein Genes ZO-1 and Occludin in Mouse Small Intestinal Epithelial Cells

The mRNA expression of ZO-1 and occludin genes was affected by α-chaconine concentration and incubation time (Table 7). In the control cells, the mRNA expression of ZO-1 and occludin genes did not change over time, whereas in the α-chaconine treated cells, the effects on the mRNA expression were concentration- and time-dependent. When α-chaconine was added at 0.4 μg/mL, the expression of ZO-1 and occludin mRNA dropped by 7–9% at 24 h, 27–30% at 48 h, and 53–55% at 72 h, respectively, compared to the control group. When the α-chaconine concentration was increased to 0.8 μg/mL, the suppression of mRNA expression was greater, being 36–44% at 24 h, 50–53% at 48 h and 63–64% at 72 h, respectively, in comparison with the control.

**TABLE 7:** Effects of α-chaconine on the mRNA expression of tight junction protein ZO-1 and occludin genes in mouse intestinal epithelial cells.

Concentration (μg/mL)	Time (h)	ZO-1 gene mRNA	Occludin gene mRNA
0	24	1.01^a^	1.05^*a*^
	48	1.02^*a*^	1.05^*a*^
	72	0.94^*a*^	0.97^*a*^
0.4	24	0.92^*a*^	0.98^*a*^
	48	0.71^*b*^	0.77^*b*^
	72	0.43^*de*^	0.46^*cd*^
0.8	24	0.65^*bc*^	0.59^*c*^
	48	0.51^*cd*^	0.49^*cd*^
	72	0.34^*f*^	0.36^*d*^
SEM		0.082	0.087
*P-*value	Conc	<0.001	<0.001
	Time	<0.001	<0.001
	Conc × Time	0.017	0.001

*The same letter on the shoulder of the mean in the same column indicates that the difference is insignificant, and the absence of the same letter means that the difference is significant. Conc, concentration. The mRNA expression of the target genes was quantified in comparison with the mRNA expression of the reference gene β-actin that was set as 1.*

### Antioxidant Ability of MODE-K Cells

The α-chaconine concentration and incubation time significantly affected the concentrations of MDA and T-GSH, the activities of GSH-Px and γ-GCS, but did not significantly affect the level of the activities of SOD and CAT (Table 8).

**TABLE 8:** Effects of α-chaconine on the antioxidant ability of mouse intestinal epithelial cells.

Concentration (μg/mL)	Time (h)	MDA content (nmol/mgprot)	T-GSH content (μ mol/gprot)	SOD activity (U/mgprot)	CAT activity (U/gHb)	GSH-Px activity (U/mgprot)	γ -GCS activity (U/gprot)
0	24	0.58^*f*^	9.80^*a*^	4.18^*a*^	548.6^*ab*^	3.21^*a*^	70.1^*a*^
	48	0.61^*ef*^	9.37^*a*^	4.23^*a*^	587.7^*a*^	3.26^*a*^	71.6^*a*^
	72	0.59^*ef*^	9.96^*a*^	4.18^*a*^	554.2^*ab*^	3.20^*a*^	71.7^*a*^
0.4	24	0.83^*e*^	9.65^*a*^	3.99^*ab*^	506.4^*b*^	2.85^*b*^	67.7^*a*^
	48	1.34^*d*^	7.42^*b*^	3.42^*bc*^	428.4^*c*^	2.36^*c*^	55.8^*b*^
	72	1.66^*c*^	5.55^*cd*^	2.68^*de*^	387.9^*c*^	1.74^*d*^	44.9^*c*^
0.8	24	1.41^*d*^	6.53^*bc*^	3.13^*cd*^	401.6^*c*^	2.06^*cd*^	54.5^*b*^
	48	1.90^*b*^	4.67^*de*^	2.29^*ef*^	369.9^*cd*^	1.28^*e*^	40.8^*c*^
	72	2.56^*a*^	3.76^*e*^	1.92^*f*^	320.4^*d*^	0.97^*e*^	28.8^*d*^
SEM		0.217	0.755	0.277	29.87	0.273	4.87
*P-*value	Conc	<0.001	<0.001	<0.001	<0.001	<0.001	<0.001
	Time	<0.001	<0.001	<0.001	0.005	<0.001	<0.001
	Conc × Time	< *x*0.001	0.004	0.053	0.057	0.001	0.004

*The same letter on the shoulder of the mean in the same column indicates that the difference is insignificant, and the absence of the same letter means that the difference is significant. MDA, malondialdehyde; T-GSH, total glutathione; SOD, superoxide dismutase; CAT, catalase; GSH-Px, glutathione peroxidase; γ-GCS, γ-glutamylcysteine synthetase; Conc, concentration.*

In the control cells, levels of measured parameters were unchanged with incubation time, but the addition of α-chaconine increased the content of MDA and decreased the levels of other measured parameters. The responses changed linearly with incubation time, but the responses to α-chaconine concentration differed in extent for different measurements. The treatment of 0.4 μg/mL α-chaconine at 24 h resulted in a decrease by 2, 5, 8, 11, and 3% for the content of T-GSH and for the activities of SOD, CAT, GSH-Px, and γ-GCS, respectively, compared with the control. When α-chaconine was added at 0.8 μg/mL, the extent of decrease in these measurements was 33, 25, 27, 36, and 22%, respectively, compared with the control.

### Expression of Anti-oxidase Genes SOD, CAT, GSH-Px, and γ-GCS

The α-chaconine concentration and incubation time on mouse intestinal epithelial cells had significant effects on the mRNA expression of SOD, CAT, GSH-Px, and γ-GCS genes (Table 9). In the control cells, the mRNA expression of these genes did not change over time gene. However, in the 0.4 μg/mL treated cells, at 24 h, exception for SOD which dropped slightly (by 6%, *P* > 0.05), the expression of all the other genes was significantly decreased (*P* < 0.05) by 18–23% compared with the control cells. The expression was further decreased by 25–30% at 48 h and by 51–61% at 72 h of incubation compared with the control cells for all the four genes. The difference between 24 and 48 h of incubation was not significant but was significantly different (*P* < 0.05) between 48 and 72 h. In the case of the 0.8 μg/mL treated cells, the mRNA expression of all genes was 31–44% lower than the control cells at 24 h of incubation. The decrease in the mRNA expression was greater than at 24 h, being 42–55% at 48 h and 57–69% at 72 h of incubation in comparison to the control cells.

**TABLE 9:** Effects of α-chaconine on the relative mRNA expression of antioxidant enzyme genes superoxide dismutase (SOD), catalase (CAT), glutathione peroxidase (GSH-Px), and γ-glutamylcysteine synthetase (γ-GCS) in mouse intestinal epithelial cells.

Concentration (μg/mL)	Time (h)	Relative mRNA expression of SOD	Relative mRNA expression of CAT	Relative mRNA expression of GSH-Px	Relative mRNA expression of γ -GCS
0	24	1.04^*a*^	1.11^*a*^	1.07^*a*^	1.07^*a*^
	48	1.06^*a*^	1.03^*a*^	1.05^*ab*^	1.00^*ab*^
	72	1.02^*a*^	1.10^*a*^	1.04^*ab*^	1.11^*a*^
0.4	24	0.98^*ab*^	0.85^*b*^	0.88^*bc*^	0.85^*bc*^
	48	0.79^*bc*^	0.75^*bc*^	0.73^*cd*^	0.74^*cd*^
	72	0.49^*d*^	0.54^*e*^	0.49^*e*^	0.43^*fg*^
0.8	24	0.72^*c*^	0.70^*cd*^	0.60^*de*^	0.67^*de*^
	48	0.62^*cd*^	0.57^*de*^	0.47^*e*^	0.54^*ef*^
	72	0.43^*d*^	0.34^*f*^	0.45^*e*^	0.35^*g*^
SEM		0.077	0.085	0.083	0.087
*P-*value	Conc	<0.001	<0.001	<0.001	<0.001
	Time	<0.001	<0.001	0.002	<0.001
	Conc × Time	0.030	0.013	0.043	0.005

*The same letter on the shoulder of the mean in the same column indicates that the difference is insignificant, and the absence of the same letter means that the difference is significant. Conc, concentration. The mRNA expression of the target genes was quantified in comparison with the mRNA expression of the reference gene β-actin that was set as 1.*

## Discussion

### Cell Proliferation, Cell Cycle, and Apoptosis

The cell proliferation measured by the MTT method can reflect the cell survival and growth ability and can also indirectly reflect the degree of epithelial cell membrane damage (Wang et al., 2017). The results obtained in this study showed that the MTT value of the cells decreased with the increase of α-chaconine concentration and the prolongation of incubation time, suggesting that α-chaconine leads to damage to cell morphology, a reduction in cell viability, and to cytotoxicity. It has been reported that α-chaconine had toxicity to intestinal crypt epithelial cells (IEC-6) and ileal cells (IEC-18) of rats and reduced the MTT values of these cells (Gee et al., 1996). It can be speculated that after α-chaconine reaches the epithelial cells, it may alter or destroy the structure of the cell in some way to affect the metabolism of the cell, and eventually lead to the blocking of cell proliferation.

The cell cycle and apoptosis processes synergize to some extent, in that some key cell cycle regulatory proteins are involved in the regulation of the apoptosis process (Xu et al., 2020). The start and the end of apoptosis are dependent on the operation of the cell cycle, and the stage of the cell cycle phase dictates the exact time when apoptosis begins during the completion of the cell cycle (Dong et al., 2019). Drug based apoptotic agents interfere with the cell proliferation cycle, inhibit the normal transformation during the cell cycle, and promote cell death (Zhang and Shi, 2011). Hence, the process of apoptosis can cause a series of changes in the structure of cell membranes, loss of mitochondrial function, degradation of proteins in cells, and changes in DNA cleavage, etc.

The cell cycle consists of two phases: mitosis (M) and mitotic intervals (G_1_, S, and G_2_), while the proliferative capacity of cells is mainly reflected in the G_2_ and S phases (Li and Xu, 2019). The increase in the length of G_0_/G_1_ phase with α-chaconine observed in this study suggests that α-chaconine can increase the number of MODE-K cells in the G_0_/G_1_ phase, resulting in more cells in DNA repair processes; while the decrease in the S phase leads to a reduction in the number of cells in DNA synthesis phases, with the decrease in the G_2_/M phase causing a decrease in the synthesis of RNA and proteins. α-Chaconine can bind to cell homogenates of various tissues, but not to DNA or RNA fragments isolated from mouse hepatocytes (Sharma et al., 1983). Thus, α-chaconine induces apoptosis in more than one way, and other ways are suggested such as by increasing the synthesis of intracellular nitric oxide to reduce the mitochondrial membrane potential or intracellular Ca^2+^ concentration for the activation of apoptotic proteins (Toyoda et al., 1991).

### Mechanical Barrier Function of MODE-K Cells

The transepithelial electrical resistance (TEER) value is an important indicator reflecting the integrity of epithelial cells and cell membrane permeability (Sheridan et al., 2020). α-Chaconine can affect intestinal permeability in mice, thereby disrupting intestinal barrier function (Patel et al., 2002). In this study, the TEER value showed an α-chaconine dose- and incubation time-dependent decrease, which may be due to an α-chaconine-induced change in the structure of the epithelial cell membrane (Friedman et al., 1996; Friedman and McDonald, 1997). This result could account for the mechanism of the *in vivo* effects (Friedman et al., 1997) which result in the alteration of the ion transepithelial transport channel, a decrease in TEER, and an increase in the permeability of the intestine, consequently causing the loss of protein and other nutrients from the intestine, and the invasion of bacteria and other antigens into the bloodstream.

Alkaline phosphatase is an endogenous enzyme in tissues and cells. Intestinal AKP is distributed in large quantities in intestinal tissues and plays an important role in maintaining the integrity of intestinal epithelial cells. Alkaline phosphatase is an important binding protein on the cell membrane and plays an important regulatory role in cell growth, viability, and metabolism (Bol-Schoenmakers et al., 2010). If AKP is lost from the intestine, the permeability of epithelial cells will increase, causing inflammation and sepsis, and other diseases (Bates et al., 2007). Therefore, the determination of extracellular AKP activity is another important indicator for the detection of cell membrane permeability and integrity. The present study showed that α-chaconine treatment enhanced the extracellular AKP activity of mouse small intestinal epithelial cells, which may be due to α-chaconine changing the cell membrane structure and further causing the increased permeability. A study showing that soybean agglutinin induces an increased AKP activity of IPEC-J2 cells also highlighted the importance of intestinal AKP in the maintenance of the integrity of intestinal epithelial cells (Pan et al., 2013).

Lactate dehydrogenase is the key enzyme for cellular energy metabolism and exists in the cell cytoplasm. The change of LDH enzyme activity in the culture medium is a very sensitive indicator of cytotoxicity (Yuan et al., 2019). Under normal physiological conditions, the LDH enzyme is present within the cell. If the cell membrane is destroyed, the LDH enzyme in the cell will leak to the outside of the cell, increasing the activity of the extracellular LDH enzyme (Xiao et al., 2020). This study found that the LDH activity of the cells increased with α-chaconine treatment in a dose- and time-dependent manner, which is consistent with the findings that LDH activity increased with the addition of α-chaconine or α- solanine into rat glioma cells (Yamashoji and Matsuda, 2013). The effect of α-chaconine on rat IEC-6 and IEC-18 cells also led to the release of intracellular LDH and the increase of LDH content in the extracellular fluid (Gee et al., 1996). A similar result was also reported with human intestinal epithelial cell line Caco-2 (Mandimika et al., 2007). These results suggest that the cell structure is damaged by α-chaconine, resulting in increased permeability of the cell membrane and impaired mechanical barrier function of the cell, and consequently leading to a large number of intracellular enzymes, possibly including apoptotic signaling molecules, entering the extracellular space and stimulating cell apoptosis. Destructive effects on the cell membrane are considered to be the most important mechanism for glycoalkaloids by which the structural and functional integrity of cells or tissues of organisms are damaged (Roddick et al., 1988; Smith et al., 2001; Nenaah, 2011). From the results of this study, we concluded that α-chaconine destroys the mechanical barrier of intestinal mucosal cells by affecting the structure of the cell membrane and the transepithelial cell electrical resistance.

The tight junction is an important structure to maintain the mechanical barrier and permeability of mucosal epithelium. Cytoplasmic protein ZO-1 and the transmembrane protein occludin are two important proteins in tight junctions (Van Itallie and Anderson, 2014). They are not only involved in regulating cell material transport and maintaining epithelial polarity, but are also related to information transmission and the regulation of cell proliferation and differentiation, gene transcription, and other processes. The presence and quantity of ZO-1 and occludin proteins reflect the mechanical barrier of the intestine. As a result, their distribution and relative gene expression are reliable indicators for the state of intestinal tight junction and the disruption of barrier function (Cai et al., 2018). The expression of ZO-1 and occludin can affect the TEER value.

The results of this study showed that the distribution and relative expression of ZO-1 and occludin in cells decreased with α-chaconine treatment in a dose- and time-dependent manner. The reduction of ZO-1 and occludin protein levels damages mainly the intestinal mucosal barrier, which is consistent with reports in the literature (Zhao et al., 2015; Yang et al., 2017).

### Cellular Antioxidant Functions

Malondialdehyde is one of the most important products in the process of membrane lipid peroxidation, which not only indicates the degree of free radical production but also reflects the degree of lipid peroxidation and indirectly suggests the degree of cell damage (Barrera et al., 2018). The content of MDA is an indicator of the degree of lipid peroxidation. Superoxide dismutase can scavenge free radicals in the body and catalyze the decomposition of peroxyl radicals to form hydrogen peroxide. Glutathione is a very important antioxidant enzyme in organisms. It can scavenge hydrogen peroxide and lipid peroxides produced in the body and block the damage caused by reactive oxygen radicals (Pastore et al., 2003). Hydrogen peroxide can be converted into water by GSH-Px (Motoori et al., 2001) so that cells are protected from oxidative damage by peroxyl radicals. The reduction in SOD enzyme activity indicates that there are more free radicals in the body to be scavenged (Goc et al., 2017). Catalase can decompose a large amount of H_2_O_2_ into water and molecular oxygen, or catalyze the reduction of H_2_O_2_ by the O^2–^ provided by the hydrogen donor molecule to prevent the primary initiation of lipid peroxidation reaction, and thus reduce the damage of organic hydroperoxides (Ighodaro and Akinloye, 2018). Glutathione peroxidase accelerates the decomposition of H_2_O_2_ into water, and specifically catalyzes the reduction reaction of reduced glutathione and H_2_O_2_, to protect the integrity and function of cell membrane structure (Zhang et al., 2020). Glutamylcysteine synthetase is a dimer composed of regulatory and catalytic subunits of glutamylcysteine ligase. It is a rate-limiting enzyme for glutathione (GSH) synthesis, while GSH can neutralize free radicals and reactive ROS.

In this study, MDA content increased with the dose of α-chaconine and incubation time. We speculate that with the action of α-chaconine, mouse small intestinal epithelial cells might produce a large number of free radicals that trigger membrane lipid peroxidation (Pisoschi and Pop, 2015), which causes damage to the cell membrane system, destroys the oxidative metabolic process of cells, and results in oxidative damage to mitochondria. The significant decrease in T-GSH level and activities of SOD, CAT, GSH-Px, and γ-GCS may be due to the consumption of various oxygen free radicals (Hayes and McLellan, 1999) with α-chaconine, which leads to an increase in the sensitivity of small intestinal epithelial cells to oxidative damage in the present study.

The study of antioxidant enzyme genes has become an important research direction. The activity of antioxidant enzymes is determined by the mRNA expression of their genes and the stability, survival, and activity of the proteins. The addition of exogenous nutrients can affect the mRNA expression of antioxidant enzyme genes in an animal (Baker et al., 1998). The results of the present study showed that the mRNA expression of SOD, CAT, GSH-Px, and γ-GCS was decreased by the treatment of cells with α-chaconine, which reduced the defense ability of the cell’s antioxidant enzyme system against ROS, leading us to conclude that α-chaconine results in oxidative damage which may be one of reasons for the apoptosis of small intestinal epithelial cells.

## Conclusion

α-Chaconine inhibited cell proliferation and promoted cell apoptosis in a dose-dependent and time-dependent manner by blocking cells in the G0/G phase and affecting DNA synthesis. α-Chaconine decreased antioxidant enzyme activity in MODE-K cells, which contributed to lipid peroxidation, cell injury, destroyed the integrity of the cell membrane structure, destroyed the mechanical barrier and permeability of the mucosal epithelium, and finally inhibited cell proliferation and accelerated cell apoptosis.

## Data Availability Statement

The original contributions presented in the study are included in the article/Supplementary Material, further inquiries can be directed to the corresponding author/s.

## Author Contributions

YH conceived and planned the study, acquired funding, and supervised all research. YH, LW, JZ, and JC collected the data. YH, XS, and XC analyzed and interpreted the data. YH and XS prepared the tables and figures, and wrote the manuscript. XS and AM revised the manuscript. All authors approved the final version of the manuscript.

## Conflict of Interest

The authors declare that the research was conducted in the absence of any commercial or financial relationships that could be construed as a potential conflict of interest.
