# Supplementary material for: α-Chaconine Affects the Apoptosis, Mechanical Barrier Function, and Antioxidant Ability of Mouse Small Intestinal Epithelial Cells
Source: Front Plant Sci. 2021 Jun 9;12:673774. doi: 10.3389/fpls.2021.673774 (PMC8220139; doi:10.3389/fpls.2021.673774)
Supplement: Supplementary file 1 [file Data_Sheet_1.docx]

**Control 0.4 μg/mL 0.8 μg/mL**

**Zo-1**
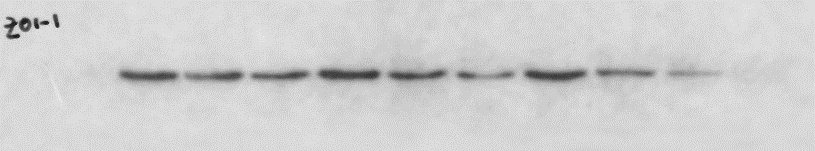


**Occluding
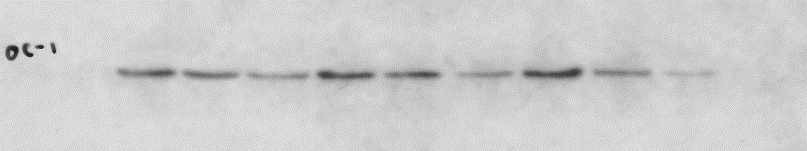
**

**β-actin** **
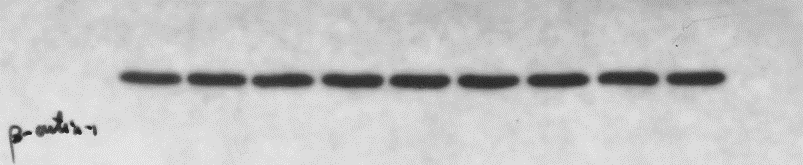
**

**24 h**

**Control 0.4 μg/mL 0.8 μg/mL**

**Zo-1** **
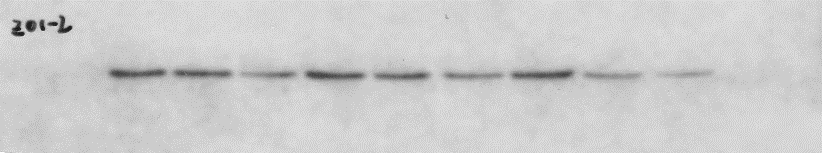
**

**Occluding** **
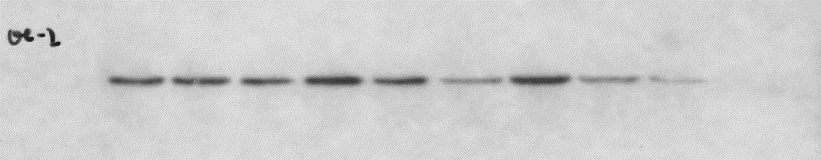
**

**β-actin** **
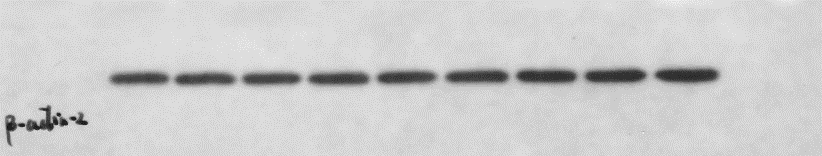
**

**48 h**

**Control 0.4 μg/mL 0.8 μg/mL**

**Zo-1** **
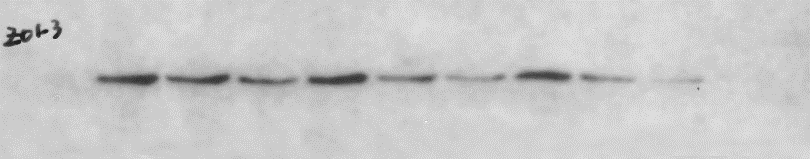
**

**Occluding** **
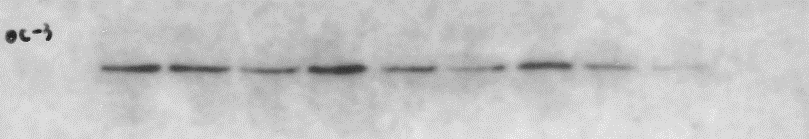
**

**β-actin** **
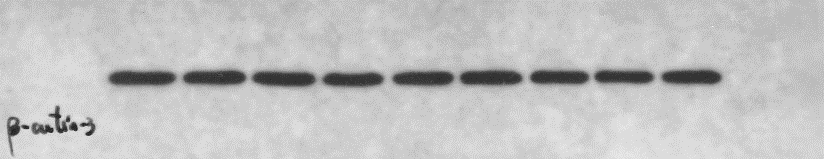
**

**72 h**

**Supplementary Figure 1. Western blotting of tight junction proteins following α-chaconine treatment. Fifteen microliters of 0 (Control), 0.4 or 0.8 µg/mL α-chaconine-treated total protein extracts were analyzed by immunoblotting with antibodies for zonula occludens-1 (ZO-1) and occludin. β-actin was used as the control. Western blots from three independent experiments are shown.**
